# Cost-effectiveness of automated digital CBT (*Daylight*) for generalized anxiety disorder: A Markov simulation model in the United States

**DOI:** 10.1371/journal.pmen.0000116

**Published:** 2024-08-26

**Authors:** Michael Darden, Jenna R. Carl, Jasper A. J. Smits, Michael W. Otto, Christopher B. Miller

**Affiliations:** 1 Carey Business School, Johns Hopkins University, Baltimore, Maryland, United States of America; 2 Big Health Inc., San Francisco, United States of America; 3 Department of Psychology, The University of Texas at Austin, Austin, Texas, United States of America; 4 Department of Psychological and Brain Sciences, Boston University, Boston, Massachusetts, United States of America; 5 Big Health Inc., London, United Kingdom; Universidad del Valle, COLOMBIA

## Abstract

This study examines the cost-effectiveness and cost-benefit of a fully automated smartphone-delivered digital cognitive behavioral therapy (CBT) intervention for Generalized Anxiety Disorder (GAD). In a simulated Markov model, 100,000 individuals with GAD were studied under one of five (n = 20,000 per arm) treatments (digital CBT [Daylight], individual CBT, group CBT, pharmacotherapy, or no GAD treatment). Model inputs were determined from the literature and included direct treatment costs and disease costs. Net monetary benefit (NMB) determined whether digital CBT is cost-beneficial from both a private payer and societal perspective in the United States in 2020. Digital CBT was found to generate the lowest 12-month total cost ($167.02m) and the second highest number of total quality-adjusted life years (14,711.86). Digital CBT showed a positive NMB relative to each alternative treatment and to no treatment for GAD in both a payer and societal perspective. Relative to no treatment, the average NMB of digital CBT was $1,836.83 from the payer perspective and $4,126.88 from the societal perspective. Digital CBT generates the most value in both a payer and societal perspective, and results were robust to sensitivity analysis with respect to effectiveness, pricing, and attrition parameters.

## Introduction

The societal economic cost of anxiety disorders is high and as early as 1990, costs in the United States already exceeded $42 billion [1] and were more than €74 billion to Europe in 2010 [2]. Direct medical expenditure alone has been estimated at approximately $33.7 billion in 2013 per year, $38 billion in 2020 US dollars [3]. Generalized Anxiety Disorder (GAD), which has the highest mean annual medical cost of all the anxiety disorders [4], is persistent and common [5], affecting approximately 4% of the US adult population [6]. GAD is debilitating and characterized by excessive anxiety and worry about a variety of events or activities that is difficult to control with impairments to psychosocial functioning [7]. The disorder is a significant public health concern and economic burden because it leads to reduced health-related quality of life (HRQoL), impaired work productivity and increased healthcare utilization [4,8,9]. Most individuals experience co-occurring mood, substance use, and anxiety disorders, as well as medical comorbidities, which can further contribute to increased healthcare utilization [10–14]. Patients are also more likely to visit a physician or specialist service than those with other anxiety disorders [15,16]. For management, patients frequently present to primary care providers [17], and the prevalence of GAD in primary care is higher than for other psychiatric disorders [15].

Both pharmacotherapy and Cognitive Behavioral Therapy (CBT) are first-line treatments for GAD in adults [18–20]. When compared with pharmacotherapy, CBT is better tolerated with lower rates of treatment attrition [21], and fewer side effects [22]. Accordingly, CBT has been recommended as the treatment of choice for GAD, though Selective Serotonin Reuptake Inhibitors (SSRIs) and Serotonin and Norepinephrine Reuptake Inhibitors (SNRIs) can be used when CBT is unavailable, ineffective, or if not preferred by patients [23]. Despite high healthcare utilization rates and costs, many patients with an anxiety disorder, however, do not receive either adequate pharmacotherapy (appropriate dose and duration) or CBT-focused psychotherapy [17]. For those who do receive treatment, the vast majority (93%) obtain medications and far less (30%) receive counseling or psychotherapy [5]. CBT is traditionally delivered by a therapist either individually or in groups [23]. Therapist-delivered CBT may be less utilized because of difficulty with access due to a shortage of trained therapists, waitlists and difficulty with distance, scheduling, costs, and a perceived stigma of therapy [17,24,25].

Fully automated (i.e., standalone without therapist input) digital CBT may help overcome barriers to access CBT because digital devices (computers, tablets, and smartphones) are ubiquitous in the US with 85% of Americans reporting access to a smartphone in 2020 [26]. Smartphone-delivered applications or ‘apps’, that satisfy rigorous evidence standards in clinical trials [27], may offer a promising safe and effective way to deliver reliable access to evidence-based CBT interventions because they permit access at any time or location [28]. *Daylight*^TM^ is one novel, smartphone-based and fully automated digital CBT intervention designed to facilitate learning of key CBT concepts and skills through real-time application. *Daylight* has been evaluated in two published clinical studies [28,29] and further research (ClinicalTrials.gov ID: NCT05748652) is underway. *Daylight* aims to deliver CBT in an engaging and accessible way through a smartphone and was developed in collaboration with clinical psychologists, filmmakers, podcast producers, and designers.

Despite the potential for automated smartphone-delivered digital CBT to deliver a scalable treatment for GAD, without therapist support, assessment of its cost-effectiveness is limited. A recent review of economic evaluations of digital mental health interventions by Jankovic and colleagues [30] highlight an overall lack of evidence regarding their cost-effectiveness, with only one study assessing a former digital therapist-guided intervention (*Lantern*) for GAD [31]. The authors of this previous study based their economic model on data from an unpublished pilot study, and suggested further research should include more robust randomized controlled trial data, which are now available [32]. It appears there is a lack of studies evaluating the cost-effectiveness of a fully automated digital CBT intervention for GAD. Previous systematic reviews suggest a lack of cost-effectiveness research in GAD populations using unguided and automated digital interventions [30,33–35]. Individual studies have evaluated the cost-effectiveness of guided-digital CBT for anxiety disorders in England [36] and Australia [37], and we are unaware of any studies that have evaluated the cost-effectiveness of a fully automated smartphone-delivered digital CBT intervention for adults (18 years and above) seeking treatment for GAD in the US.

In a simulated Markov model, we examine the cost-effectiveness (using direct treatment costs and disease cost estimates) and cost-benefit (quantified by the overall net monetary benefit: NMB), of fully automated digital CBT for GAD relative to the opportunity cost of no treatment for GAD (i.e., a do-nothing scenario), using both a private payer and societal perspective over two 6-month cycles across a 12-month time horizon. Examples of private payers are self-insuring large employers or private health insurance companies. These actors care about the direct costs of treatment; the implications of disease on expenditures (e.g., health care expenditures, and value of employee time). A cost-effectiveness assessment is important because it allows payers to make decisions about the allocation of resources, and in this case, a payer may be an insurance company, employer, or health system. The social perspective includes these considerations, and it adds the innate value of health improvement. For relative comparisons, we also consider therapist-delivered individual and group-based CBT, and pharmacotherapy for GAD (each compared with no treatment for GAD). A Markov model is a useful analytical framework and is widely used to help guide decision making for payers in economic evaluations of healthcare interventions [38]. Markov models capture transitions between health states in response to treatment (e.g., if someone moves from having an anxiety disorder to a state of remission). Models are based on relevant assumptions from the literature, including direct and indirect cost estimates, treatment remission probabilities, and quality of life assessments over time.

## Materials and methods

We used a Markov model to examine the potential cost-effectiveness and cost-benefit of automated digital CBT [*Daylight*^TM^, developed by Big Health Inc. [39] for GAD from a private payer and societal perspective. Cost-effectiveness was evaluated by summing the total treatment costs (both direct and indirect), the costs of disease (health care expenditures, and value of employee time) and the total quality-adjusted life years (QALYs) generated over one year. We define direct treatment costs as those required to undergo treatment. For example, the direct costs of digital CBT include a one-time payment of $400 for access. For pharmacotherapy, we include costs of physician visits and prescription refills. In addition to these costs, we consider the costs associated with GAD, including additional healthcare utilization and work disability days. Both payer and societal perspectives consider these costs, but what differentiates these perspectives is the innate value of health improvement, which we define via health utilities and QALYS. QALYs are defined as being in a given health state over one year and range from zero (deceased) to one (perfect health). They are the product of healthy utility, measured by HRQoL, multiplied by one year of life [40]. Cost-benefit was measured using the NMB [41]. The NMB quantifies all of the costs and all of the benefits as a dollar amount, and a positive NMB indicates digital CBT is cost-beneficial relative to an alternative treatment for GAD. We present two NMB specifications in the model. The payer NMB, which we conceive of as a self-insuring employer who is concerned with both worker health expenditures and worker productivity, includes the costs of treatment and the savings associated with reduced costs of GAD-related healthcare expenditure and work disability days. The payer perspective does not include health utility gains measured by QALYs. For the societal perspective, we added health utility gains of GAD remission to the payer NMB. Health utility gains were monetized, and we valued one QALY at $50,000 [42]. We did not conduct sensitivity analysis on the assumed value of a QALY because our goal with the societal perspective was to demonstrate that simply valuing the health gains at any level adds to the NMB determined in the payer perspective.

**Fig 1:**
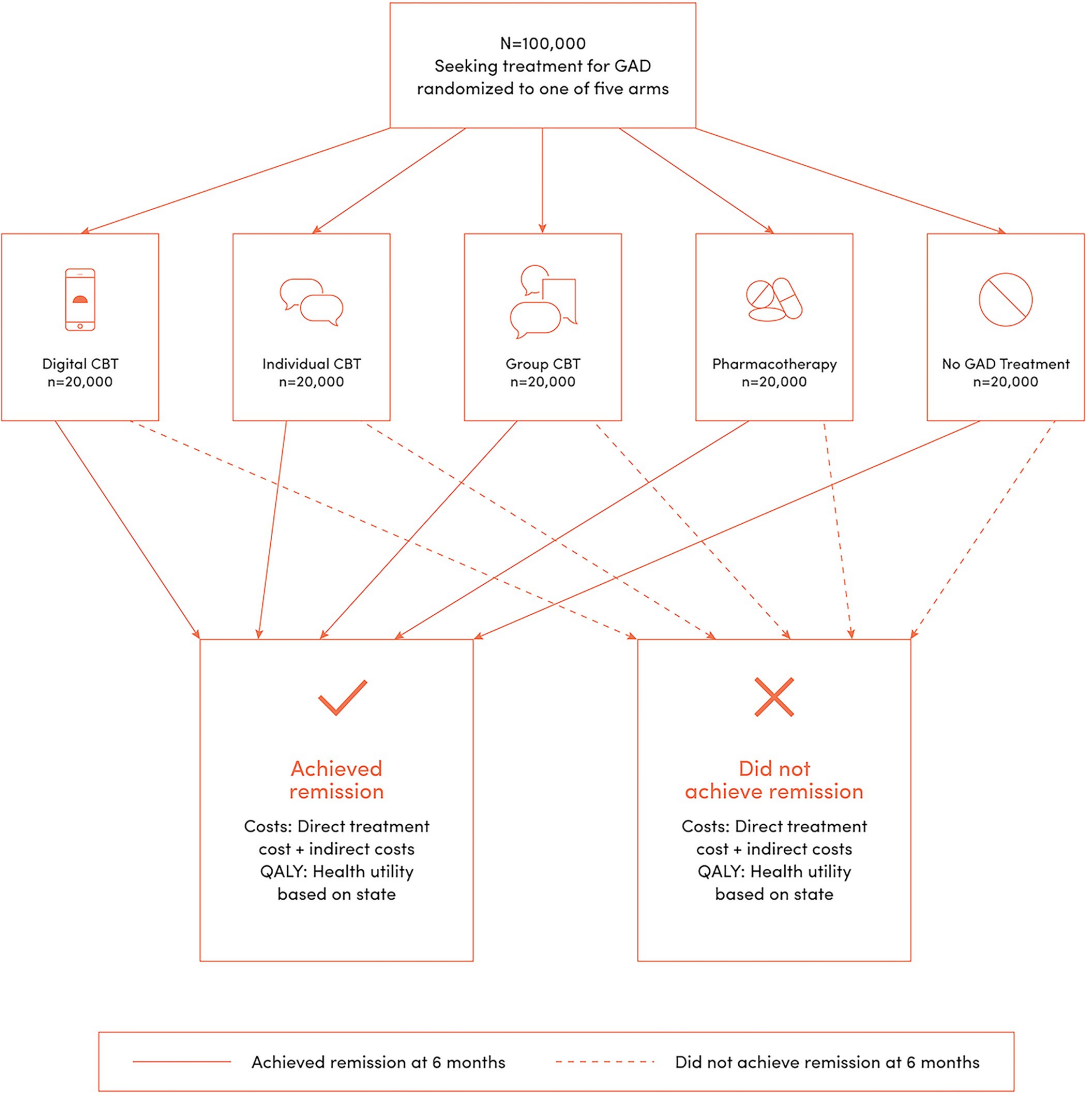
The figure demonstrates the treatments, states, outcomes, and costs, both direct and indirect of the Markov Model across the 6-month treatment time horizon. CBT: Cognitive Behavioral Therapy; QALY: Quality-adjusted life year.

The flowchart in Fig 1 illustrates the initial 6-month treatment time frame for this model. The model simulates five GAD treatment options using a cohort of 100,000 individuals with clinically defined moderate-to-severe symptoms of GAD seeking treatment in the US in 2020. In our cost-benefit analysis, we compare digital CBT to alternative treatments including individual and group CBT (delivered by a therapist) and pharmacotherapy for GAD. Simulated individuals were blocked into five treatment arms (*n* = 20,000 per arm) of either: (1) digital CBT (*Daylight*), (2) individual CBT, (3) group CBT, (4) pharmacotherapy, or (5) no GAD treatment. The time horizon for this model was 12-months and all treatment costs, GAD associated costs, and treatment benefits were summed over two 6-month cycles. A 12-month time horizon is consistent with previous models in anxiety disorders [35]. The model was calibrated using key inputs from the literature, summarized in Table 1, using Stata [43] with standard techniques for simulation methods [44]. For any dollar amount, we included an annual discount rate of 3%.

**Table 1:** Basic parameter assumptions for the simulated Markov model.

Parameter Name	Value assumed	Source
**Remission probabilities**		
Digital CBT	60%	Carl et al. (2020) [28]
Individual CBT	60%	GAD remission Springer et al. (2018) [45]
Group CBT	60%	GAD remission Springer et al. (2018) [45]
Pharmacotherapy	50%	Baldwin et al. (2011) [46]
No GAD treatment	15%	Hunot et al. (2007) [47] Yonkers et al. (2003) [48]
**Costs of Treatment**		
Digital CBT (*Daylight*)	$400.00	Big Health, Inc. [39]
Individual CBT	$2,788.50	The Current Procedural Terminology code set 90837 + 43% Private payer rate [49]
Group CBT	$579.15	The Current Procedural Terminology code 90853 + 43% Private payer rate [49]
Pharmacotherapy	$236.69	Medicaid (2020) and Health Care Cost Institute [50].
No GAD treatment	$0.00	Assumption
**Disease costs of GAD**		
Healthcare Expenditure	Physician Office Visit: $195.25 None: 2.4 Visits GAD: 4.4 Visits ER Visit: $1,004.70 None: 2.8% GAD: 5% Hospitalization: $5,100*1.22 = $5,722 None: 2.8% GAD: 5%	Health Care Cost Institute (2018) [50]. Hargraves & Kennedy, (2019). [51] Owens et al., (2019) HCUP Statistical Brief: Average Cost of Hospitalization for Anxiety (see Appendix A). [52]
Disability Days (median daily wage) to capture loss of work productivity due to absences and impaired work performance with GAD.	$206 None: 7.8 Days GAD: 21.4 Days	US Bureau of Labor Statistics (2020). [53] Assumes a 40 hour week.
**Health Utility**		
GAD quality-adjusted life years	None: 0.8 Moderate-to-severe GAD: 0.66	Kumar et al. (2018). [31]
**Attrition Rate**		
Digital CBT	16%	Carl et al. (2020). [28]
Individual CBT	9%	Hunot et al. (2007). [47]
Group CBT	24%	Hunot et al. (2007). [47]
Pharmacotherapy	30%	Sheehan et al. (2008). [54]
**Median Hourly Wage**	$27.72	US Bureau of Labor Statistics (2020). [53]

Note: CBT: Cognitive Behavioral Therapy; ER: Emergency Room; GAD: Generalized Anxiety Disorder. All values are across 6-months and costs are quantified in 2020 US dollars using the medical Consumer Price Index.

### Markov simulation model

Table 1 presents the key parameters from the literature that govern GAD state remission probabilities, treatment costs, disease costs, health utilities, and treatment attrition used to simulate the model. The table includes the range of estimates and the source for these assumptions. Each parameter assumes a 6-month time horizon. The following sections describe the model assumptions from the current literature.

### Generalized anxiety disorder state transition probabilities for remission

We assumed 100,000 simulated individuals seeking treatment exhibit moderate-to-severe GAD at baseline (defined as scores of ≥10 using the 7‐item generalized anxiety disorder questionnaire [GAD‐7 [5]]. We limited our Markov model to either GAD or healthy (GAD remission), and the timing of remission was randomized within each of the two 6-month cycles. We assumed that, upon achieving remission from GAD, an individual remained healthy for the remainder of their simulation (i.e., no relapse). Remission from GAD was defined using probabilities from standardized outcome measures (e.g., the GAD-7 or the Hamilton Anxiety Rating Scale), which varied by treatment arm. A range of sources were used to inform remission probabilities, and these are summarized in Table 1.

For the initial 6-month treatment cycle, the baseline model assumed a GAD specific remission probability of 60% for CBT, encompassing digital CBT and therapist-delivered individual and group based CBT. This assumption was informed by the findings of Springer et al. (2018)[45], where the authors observed that most studies on GAD reported a remission rate of 56%. To adopt a conservative approach in evaluating digital CBT relative to other CBT modalities, a slightly higher remission probability of 60% for CBT was assumed in the baseline model. The digital CBT intervention, *Daylight*, was evaluated for efficacy in a randomized controlled trial and observed a 71% remission rate [28]. For pharmacotherapy, we assumed a 50% remission rate [46]. We assumed a 15% probability for natural remission from GAD for those who did not receive any treatment, informed by waitlist or treatment as usual control conditions in a systematic review [47], and by longitudinal follow-up data from patients with GAD [48]. The model employed two 6-month cycles to project results out to 12-months. To do so, we assumed GAD remission probabilities for all treatment arms in the second 6-months reverted to the initial no GAD treatment arm probability (15%) for those who had not already achieved remission in the first 6-months. This is because both digital and therapist-delivered CBT treatments were assumed to end, in line with treatment guidance [18]. In Table 2, we report the mean QALY level as the weighted average of health utility by time in each state [55] for each treatment arm. A sensitivity analysis is used to explore how large remission needs to be for digital CBT to achieve a similar cost-effectiveness to other treatments for GAD.

**Table 2:** Total costs and QALYs for each treatment option from 100,000 simulated individuals with generalized anxiety disorder over 12-months.

Treatment (n = 20,000 per arm)	12-Month Total Cost (Millions $)	Total QALYs Generated
Digital CBT	167.02	14,711.86
Individual CBT	204.64	14,806.01
Group CBT	176.51	14,535.74
Pharmacotherapy	189.13	14,247.74
No Treatment	203.76	13,795.84

Note: Each treatment included 20,000 individuals. The total cost column includes total costs (treatment and disease costs). CBT: Cognitive Behavioral Therapy; NMB: Net Monetary Benefit; QALY: Quality Adjusted Life Year.

### Costs of GAD treatment

In line with our payer perspective, the cost of automated digital CBT (*Daylight*) for GAD was modeled as a one-time payment of $400 for 12-months access (not available direct-to-consumer). For therapist-delivered CBT, a Cochrane review identified that most studies used 15 CBT sessions lasting from 45 minutes to 2 hours [47]. For individual CBT, we therefore assumed a national median cost of $130 per session [56] for 15 (53–60 minute) sessions based on Medicare CPT Code 90837 [57]. To this price, we added a conservative 43% to reflect a private payer cost [49] to equal a total of $2,788.50 for each individual. For group CBT, we assumed a national cost of $27 for 15 (45–60 minute) sessions based on Medicare CPT Code 90853 (CPT Code 90853, [58]) plus 43% for a private payer [49] to equal a total of $579.15 for each individual. SSRIs and SNRIs are considered a first-line treatment for GAD. Escitalopram is a common SSRI routinely used for GAD management [59,60], and we defined pharmacotherapy as a daily one pill dose of generic escitalopram (10 mg) at $0.161 per day, based on the Medicaid Federal Upper Limit (*Federal Upper Limit | Medicaid*, [61]) for 180 days plus 43% for a private payer [49]. To this cost, we added the cost of one physician office visit for management (e.g., to give medication advice, review for safety, adherence and address any concerns), at $195.25 per visit from the Health Care Cost Institute [50] to equal a total of $236.69 for each individual. The medical CPI was used to scale cost figures to 2020 dollars [62]: see Table 1. A sensitivity analysis is also used to explore how the price of digital CBT impacts the cost-effectiveness relative to other treatments for GAD.

### Disease costs of GAD

Costs associated with GAD were included in this model. First, we identified excess healthcare expenditures known to be higher in those with GAD [3,4,9,12,63]. These comprise increased physician office visits and increased probabilities of Emergency Room (ER) visits and hospitalizations. As outlined in Table 1, we modeled the increased probability of ER visits and hospitalizations as a function of GAD state. Because our model resulted in differences in state transitions, it also produced variation in these health care outcomes. We then converted the mean cost of physician office and ER visits and hospitalizations to 2020 dollars using the medical CPI [62]. Second, we include absenteeism (i.e., loss of productivity due to work absences) and presenteeism (i.e., impaired work productivity) as work-related costs known to be associated with GAD. Work costs were captured through disability days, and we assume a disability day was associated with a loss of the median daily wage [4,8,9,64]. In Table 2, we report results for the mean cost, which is the average treatment and disease costs over time for each treatment arm.

### Health gains associated with GAD treatment

A range of HRQoL measures have been used across studies to inform estimates of health gains associated with GAD and remission from GAD. Following Kumar et al., (2018) [31], we assume GAD was associated with a health utility in the US of 0.66 and remission from GAD (normal health) was 0.8. This was then converted into a QALY, which is a function of the amount of time in the state of the health utility. In the initial 6-month treatment time-horizon, an individual’s QALY contribution was the amount of time with GAD multiplied by 0.66 plus the time in remission multiplied by 0.8, the assumed average health utility in normal health. For the second 6-month time-horizon, participants either achieved a state of remission from GAD based on whether they had already achieved remission in the first 6-months of CBT treatment. Participants who did not remit in the first 6-months were able to remit during the second 6-months assuming the same 15% natural remission from the no GAD treatment arm [47,48]. Timing for remission was randomized within each 6-month time-horizon and participants who remitted from GAD, at any time, always then remained in the remission state with a health utility associated with normal health (0.8).

### Treatment attrition

To account for GAD treatment attrition, the baseline simulation assumed differential attrition rates across treatments informed from trial and systematic review data. In the baseline model, we assume a 16% rate of attrition [28] for digital CBT. From Hunot et al., (2007) [47], we assumed a 9% attrition rate for individual CBT and 24% for group CBT. We assume a 30% rate of attrition for pharmacotherapy informed from Sheehan et al., (2008) [54]. We additionally provide a sensitivity analysis that varies the rate of attrition from digital CBT to show how large digital CBT attrition needs to be to achieve a similar cost-effectiveness to other treatments for GAD. Simulated participants were assumed to randomly leave treatment at any time during the first 6-months of study, they are assumed to not remit from GAD. Attrition from digital CBT, therefore, implies the full treatment and disease costs because GAD does not improve. For therapist-delivered individual and group CBT, we assume attrition occurs such that only 1/6th (two sessions) of the treatment costs are incurred and all of the disease costs. Attrition from pharmacotherapy implies an individual incurs 1/6th of the treatment costs and all disease costs. After the first 6-months of treatment, we then assume that those who leave treatment may then achieve remission according to the no treatment state transition probability of 15% for the remaining time horizon.

### Sensitivity analysis

We evaluate in what way different model parameter assumptions influence the digital CBT results from our model for both perspectives (payer and societal). First, we varied our remission assumption for digital CBT, holding other parameters constant, to determine how the NMB of digital CBT varied with its effectiveness. Second, we varied the direct price of digital CBT, holding the costs of therapist-delivered CBT and pharmacotherapy constant, to find at what price digital CBT has a positive NMB. Third, we varied the rate of attrition for digital CBT to understand how sensitive the NMB is to treatment attrition.

## Results

The total costs (treatment plus disease) and total QALYs generated after 12-months from our baseline model of 100,000 simulated individuals seeking treatment for GAD are presented in Table 2 by treatment. In this model, we include two health states (GAD or no GAD). Because our time frame is 12-months, we measure health as the weighted average health utility in each state within the year, which gives our health measure the interpretation of a QALY. Despite higher treatment costs relative to pharmacotherapy, costs are lowest for digital CBT ($167.02m) as digital CBT is assumed to be more effective than pharmacotherapy, which highlights the importance of disease costs for both payer and society. The three CBT treatment arms (digital, individual and group) result in similar total QALYs generated over 12-months (range: from 14,535.74 to 14,806.01), followed by pharmacotherapy (14,247.74), and then by no treatment for GAD (13,795.84).

### Cost-benefit of GAD treatments

Table 3 provides cost-benefit (NMB) results for digital CBT relative to each alternative treatment and no treatment for GAD from both the payer and societal perspectives. Relative to each alternative, digital CBT had a positive NMB in both perspectives. From the payer perspective, the mean NMBs of digital CBT were $1,880.97, $1,836.83, $1,105.65, and $474.39 relative to individual CBT, no treatment, pharmacotherapy, and group CBT. When incorporating the value of the intrinsic health gains in the societal perspective, under the assumption that a QALY is valued at $50,000, the ranking of highest NMB for digital CBT relative to each alternative changes to no treatment ($4,126.88), pharmacotherapy ($2,263.45), individual CBT ($1,645.60), and group CBT ($914.68). For the alternative treatments and relative to no treatment in the payer perspective, the NMB for individual, group, and pharmacotherapy was -$44.14, $1,362.45, and $731.18 and increased in the societal perspective, to $2,481.29, $3,212.20, and $1,863.43, respectively. Results therefore suggest that digital CBT has the highest NMB per individual from both the payer ($1,836.83) and societal perspectives ($4,126.88) relative to no treatment. Together, results imply that digital CBT generates the most value as an intervention to both private and public payers.

**Table 3:** Mean net monetary benefit for simulated individuals with generalized anxiety disorder over 12-months.

Digital CBT relative to:	Mean payer NMB QALY = $0 ($)	Mean societal NMB QALY = $50k ($)
Individual CBT	1,880.97	1,645.60
Group CBT	474.39	914.68
Pharmacotherapy	1,105.65	2,263.45
No Treatment	1,836.83	4,126.88

Note: Each treatment included 20,000 individuals. CBT: Cognitive Behavioural Therapy; NMB: Net Monetary Benefit; QALY: Quality Adjusted Life Year. The positive NMB for Digital CBT relative to no treatment reflects the savings from disease costs associated with improved health in the payer perspective.

### Sensitivity analysis results

**Fig 2:**
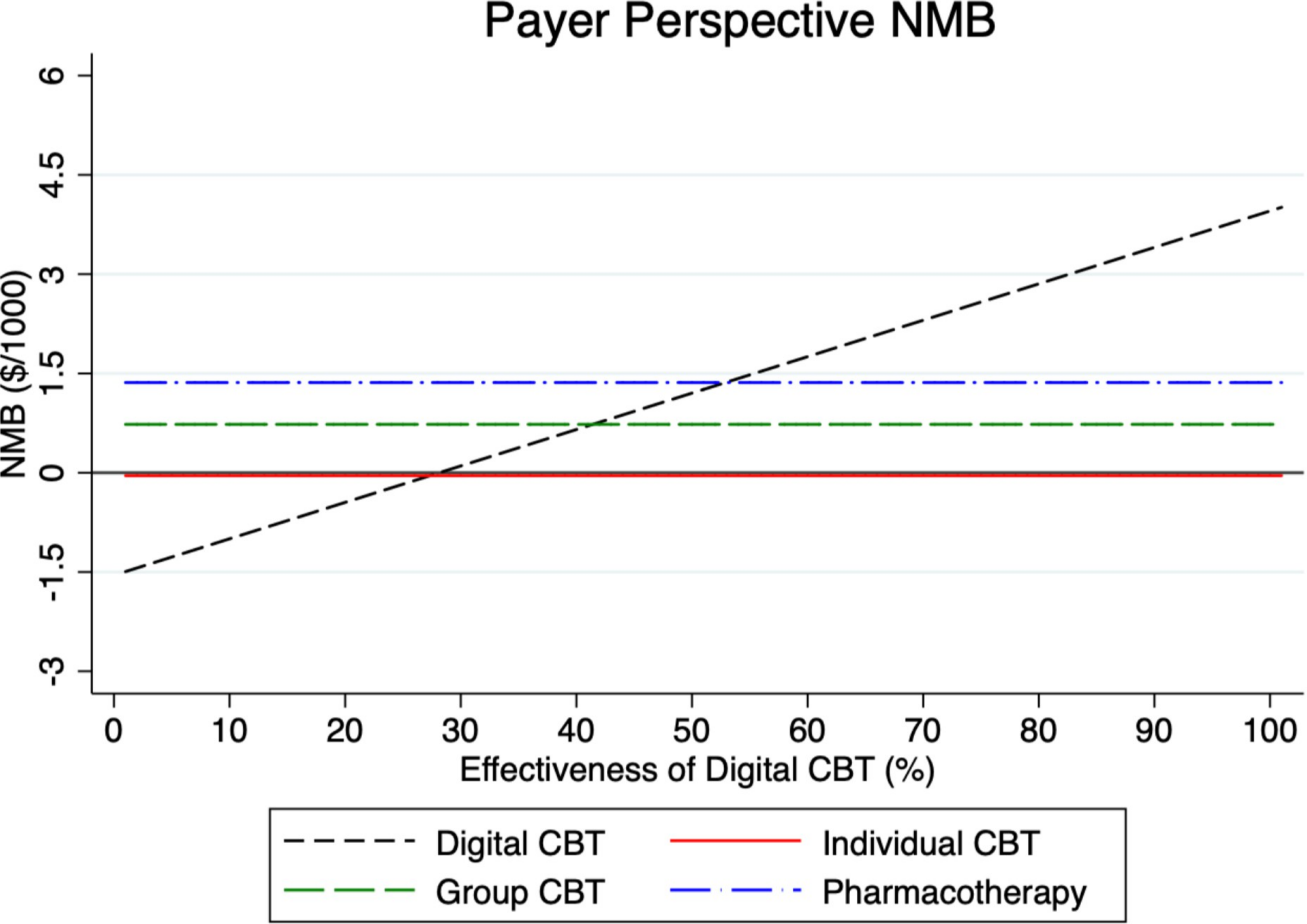
NMB by digital CBT effectiveness for the payer perspective. The figure shows that digital CBT is cost-beneficial when it is at least 28% effective. The other treatments are included with fixed effectiveness rates for reference. NMB: Net Monetary Benefit.

**Fig 3:**
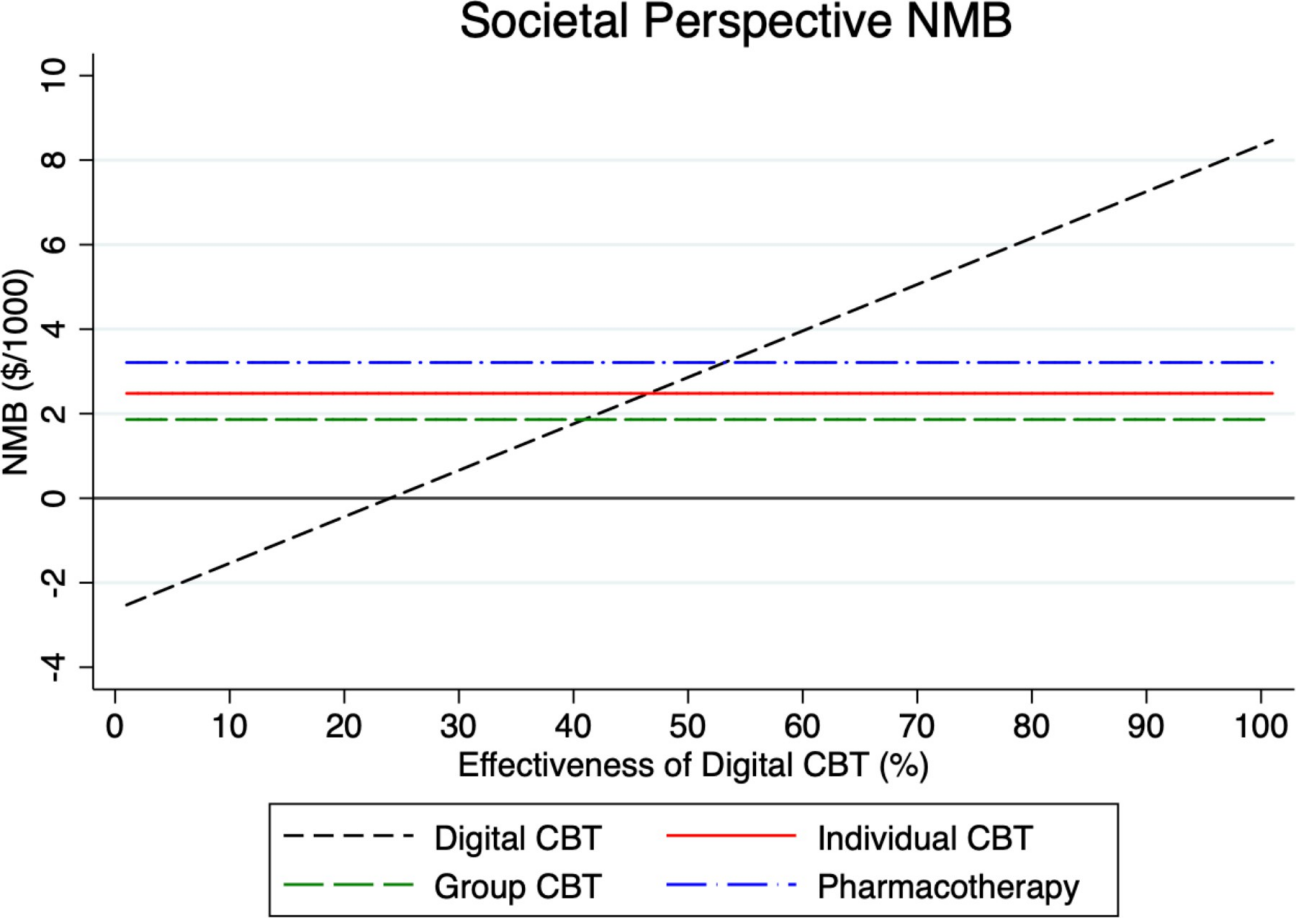
NMB by digital CBT effectiveness for the societal perspective. The figure shows that digital CBT is cost-beneficial when it is at least 24% effective in a societal perspective. The other treatments are included with fixed effectiveness rates for reference. NMB: Net Monetary Benefit.

**Fig 4:**
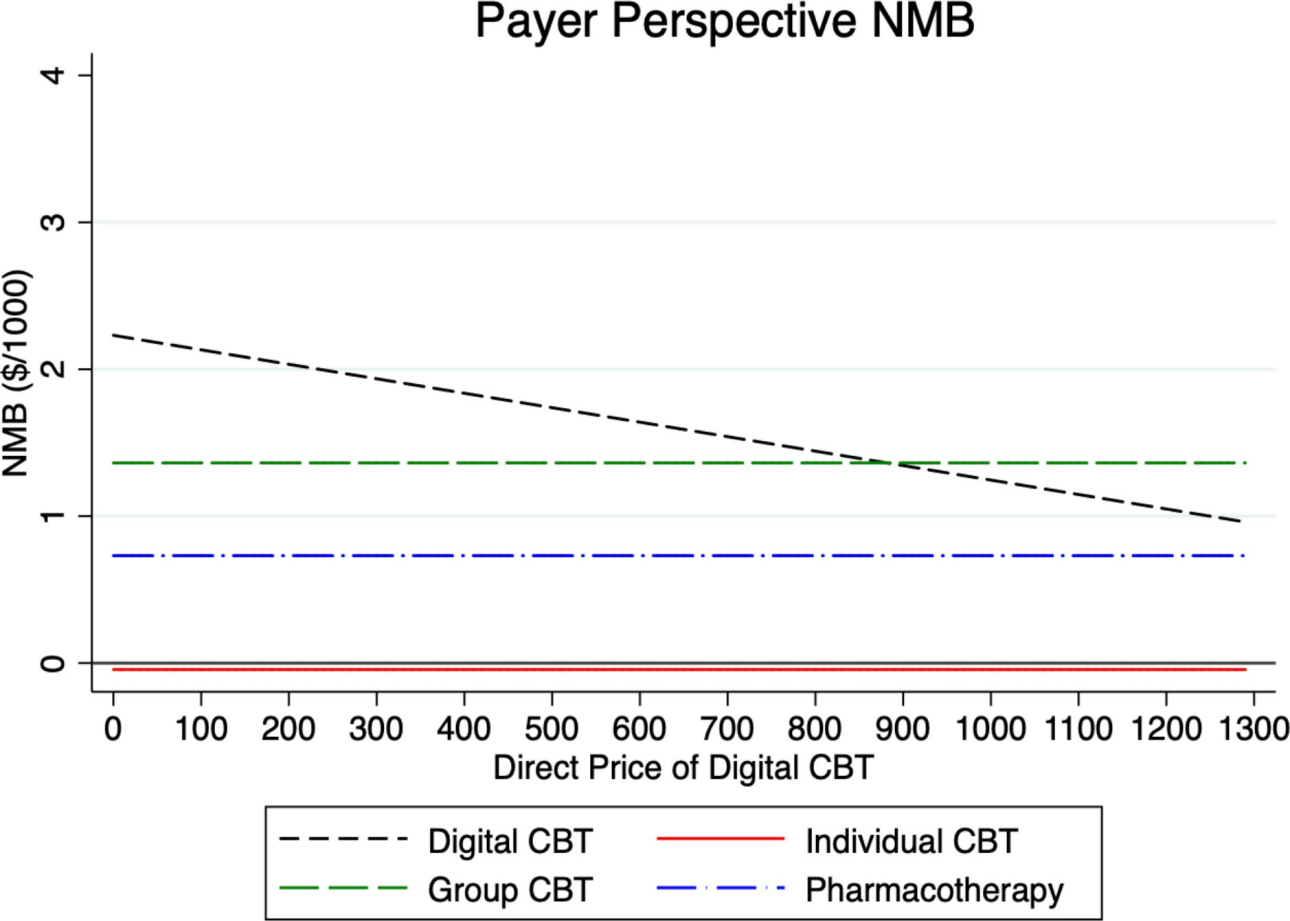
The figure plots the NMB by the price of digital CBT for the payer perspective. The figure shows that the NMB for digital CBT is cost-beneficial at any price displayed in the chart. The other treatments are included with a fixed price for reference. NMB: Net Monetary Benefit.

**Fig 5:**
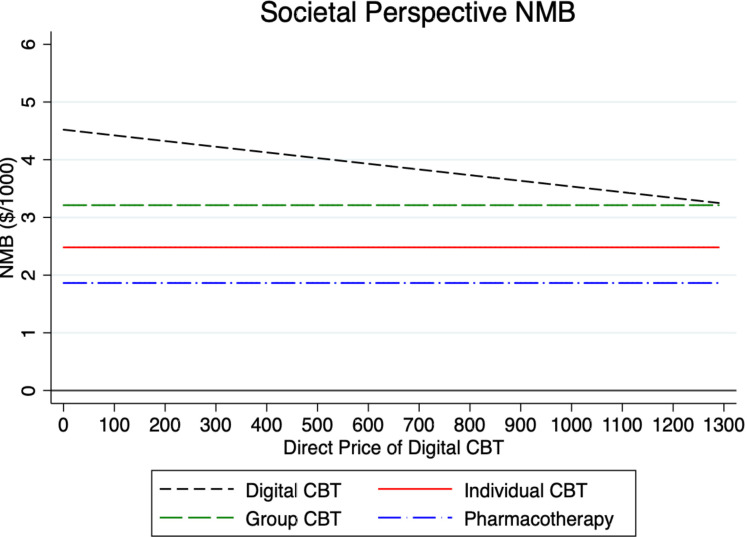
The figure plots the NMB by the price of digital CBT for the societal perspective. The figure shows that the NMB for digital CBT is cost-beneficial at any price displayed in the chart. The other treatments are included with a fixed price for reference. NMB: Net Monetary Benefit.

**Fig 6:**
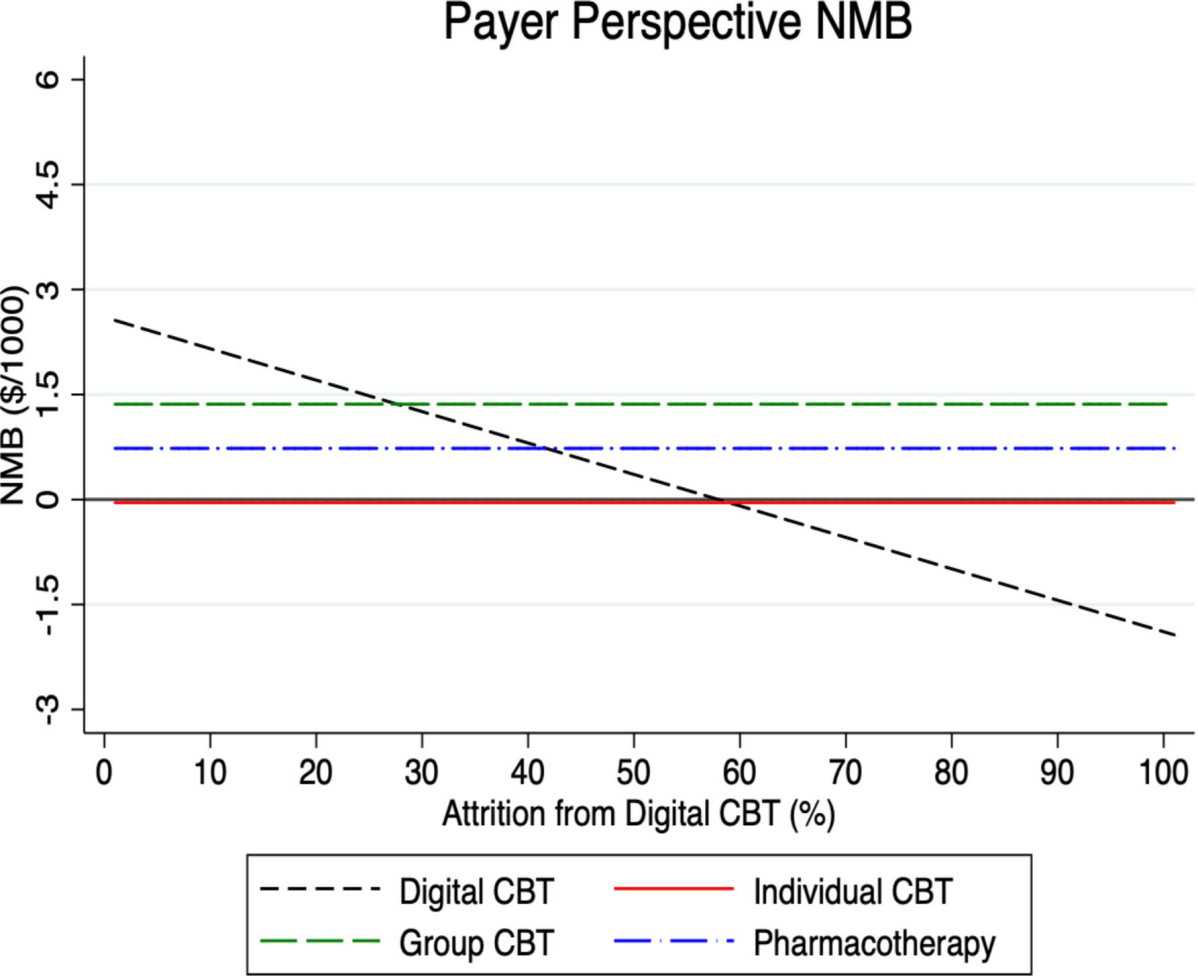
The figure demonstrates in what way the NMB of digital CBT varies by its rate of attrition in the payer perspective. The figure shows that the NMB for digital CBT is cost-beneficial at any rate of attrition up to 59%. The other treatments are included with a fixed rate of attrition for reference. NMB: Net Monetary Benefit.

**Fig 7:**
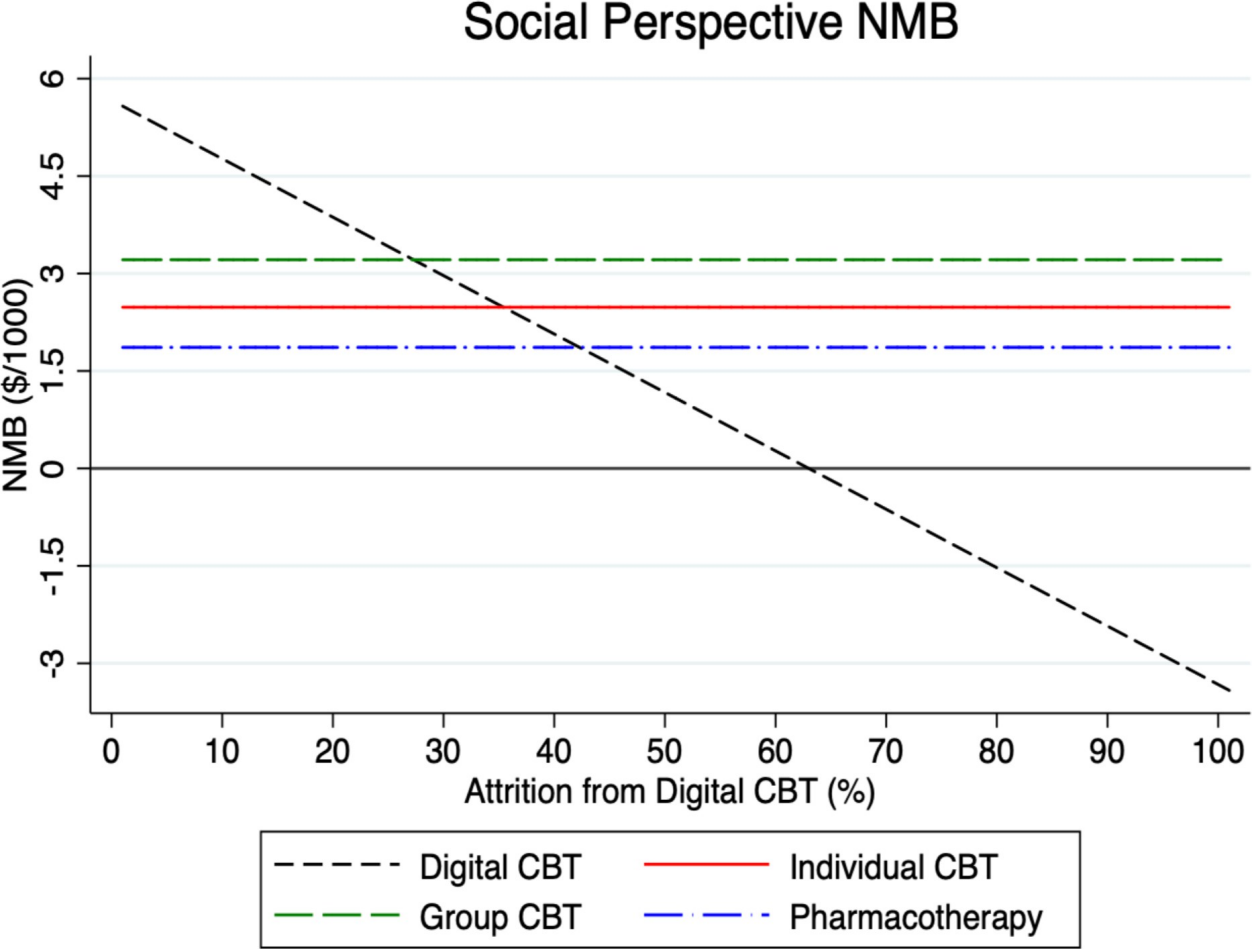
The figure demonstrates in what way the NMB of digital CBT varies by its rate of attrition in the societal perspective. The figure shows that the NMB for digital CBT is cost-beneficial at any rate of attrition up to 62%. The other treatments are included with a fixed rate of attrition for reference. NMB: Net Monetary Benefit.

To test the sensitivity of our model results, we examined in what way the NMB for digital CBT varies under different parameter assumptions in Figs 2–7 for both a payer and societal perspective. In each figure, the diagonal dashed line shows in what way the NMB for digital CBT relative to no treatment varies for each parameter. First, in Figs 2 and 3 we examined the NMB while varying the effectiveness of digital CBT, and in doing so, we evaluated the effectiveness level at which digital CBT is cost-beneficial (i.e., a NMB greater than zero) relative to no treatment. We also include individual and group CBT and pharmacotherapy (with fixed rates) relative to no treatment to compare the NMB at different levels of effectiveness of digital CBT. Digital CBT has a positive NMB when the rate of remission is at least 28% in a payer perspective (Fig 2) and at least 24% in a societal perspective (Fig 3). We assumed a baseline remission rate of 60% for digital CBT. In Figs 4 and 5, we vary the assumed direct baseline price ($400) of digital CBT to show in what way the NMB is sensitive to price in both perspectives. Digital CBT has a positive NMB at any price displayed in the chart and gives the highest NMB of all GAD treatments when priced below $890 in the payer perspective (Fig 4) and $1,300 in the societal perspective (Fig 5). Figs 6 and 7 illustrate that digital CBT can withstand a high rate of attrition, up to 59% in the payer (Fig 6) and 62% in the societal perspective (Fig 7) and remain cost-beneficial. In the baseline model we assumed a 16% attrition rate.

## Discussion

Using a simulated Markov model, we conducted a cost-effectiveness and cost-benefit analysis of a fully automated digital CBT (*Daylight*) intervention for adults seeking treatment for moderate-to-severe symptoms of GAD in the US in 2020. Digital CBT was cost-effective through lower healthcare expenditure and lower work-related costs (absenteeism and presenteeism) because of the absence of GAD. Digital CBT was cost-beneficial, and relative to other treatments was the most cost-beneficial treatment. Therefore, the cost of treating GAD with digital CBT is less than the opportunity cost of not treating GAD (i.e., a do-nothing scenario) from the perspective of both a payer and society. This result was insensitive to the assumed value of a QALY.

Fully automated digital CBT provides a cost-beneficial solution when the aim is to provide access to a guideline CBT intervention for GAD at a population level. Previous work has found CBT to be underutilized relative to pharmacotherapy [5]. Therapist-delivered CBT is difficult to access because of barriers including a lack of trained therapists, waitlists, a perceived stigma of therapy and treatment costs [17,24,25]. Digital CBT generated a positive NMB relative to individual and group CBT, pharmacotherapy, and no treatment for GAD in both a payer and societal perspective. This means digital CBT generates the most value. Treatment saves money by reducing the significant disease costs associated with GAD relating to increased healthcare expenditure, work-related costs and impaired health utility compared with no treatment for GAD. When we monetized QALY gains associated with improvement in GAD, digital CBT is the cheapest alternative, even relative to no treatment. This is because the lower average 12-month cost of digital CBT results in more QALYs per dollar than what can be achieved with individual and group CBT, and pharmacotherapy. From a payer perspective, results show investments in anxiety treatments pay for themselves by reducing healthcare and work-related costs common with GAD [3]. From a societal perspective, the NMB for digital CBT is larger because of the additional health gains associated with GAD remission, which is valued through improvements in health utilities, measured by gains in QALYs.

Sensitivity analyses were used to explore, in what way, changes to our baseline model assumptions regarding treatment effectiveness, price and attrition impact the payer and societal NMB results. Across both perspectives, digital CBT remains cost-beneficial (positive value for NMB) when it is 28% effective in the payer perspective (Fig 2) and 24% effective in the societal perspective (Fig 3). The available evidence indicates that the digital CBT under study has a remission rate up to 71% [28]. In Figs 4 and 5, we find the NMB is not overly sensitive to the direct price of digital CBT. This is because digital CBT has a positive dollar value NMB at any price displayed in either the payer or societal perspective. All treatments are found to be cost-beneficial and do not reach a $0 NMB value in either Fig 4 or Fig 5, irrespective of price. When digital CBT is priced at $400 for 12-months access to the full program, both therapist-delivered CBT and pharmacotherapy must be more effective than digital to give an equivalent NMB. This is because the digital CBT has the lowest treatment cost. Digital CBT is also able to withstand a high rate of treatment attrition and remain cost-beneficial (up to 59% in the payer perspective [Fig 6] and 62% in the societal perspective [Fig 7]).

A strength of this paper is the inclusion of both costs of treatment and disease costs of GAD (healthcare expenditure and work-related costs) in both a payer and societal (by including monetized health utilities) perspective. The use of both direct and indirect costs is like previous work [31,65], and allows us to capture the potential treatment benefits of remission from GAD in different perspectives. For payers, the inclusion of indirect costs is important because they (e.g., employers who provide health plans to employees in the US) are ultimately responsible for excess healthcare and work-related costs resulting from those who do not achieve remission from a more cost-effective treatment. From the societal perspective, indirect costs are important because better productivity adds value throughout an economy. For individuals, cost-effective treatment contributes to better health gains and lower healthcare utilization over time. Fully automated digital CBT provides a scalable treatment without the need for a therapist, and this provides additional benefits for individuals that were not quantified in the model including anytime access from any location [29]. We did not include travel-related costs for office-based visits or forgone wages for either in-person or telehealth appointments that may occur during a workday. Previous work has evaluated the cost-effectiveness of digital therapist-guided CBT for GAD and found it to be cost-saving [31]. Telehealth removes the geographical barrier for accessing CBT in-person with a therapist and saves transportation costs for individuals but not for payers. Unlike automated digital CBT, telehealth still requires trained clinicians to deliver CBT at a scheduled time. Fully automated digital CBT is a more scalable cost-effective solution as it permits access to CBT at any time or location without a wait list. These model results may also understate the wider benefits from CBT when we consider the safety profile of treatments. This is because people who use CBT for GAD may be less likely to experience costs relating to harms from pharmacotherapy [22], and healthcare costs have been found to increase in the year following pharmacotherapy management for GAD [60]. Our 12-month model conservatively assumes costs from pharmacotherapy end after 6-months of treatment with a relatively high rate of remission (50%). In reality, ongoing medication management can persist beyond the 12-month time horizon used in this study [66]. Therefore, a strength of this model is that results are conservative because we omitted costs that may have further benefited digital CBT and because we were generous with alternative treatment assumptions.

Our study has several limitations, and it is important to note that results here are based on a simulated Markov model and not from real-world patient data. Health economic models are typically used to guide decision making for payers when data are difficult to obtain or not available. Future work should now evaluate cost-effectiveness using real-world patient data over a longer time-horizon. We were unable to undertake robustness testing because of a lack of variability in the published data concerning costs associated with GAD compared with those without. The model was simplistic because of our dichotomous end state of GAD or remission. It is difficult to quantify linear parameter estimates for GAD improvement from the literature. We assumed different rates of remission for CBT and pharmacotherapy, and this was informed by the literature and not by head-to-head trials. Our model also assumes a homogenous sample of individuals, and this implies that we were unable to explore heterogeneity in cost-effectiveness. This Markov model approach is similar to what would occur if a sample of GAD patients were allocated to treatments randomly in a clinical trial. A potential limitation is that the patient sample in this model may be relatively homogeneous, which may not adequately represent the diverse characteristics and comorbidities present in real-world GAD populations.

We must assume a homogenous sample because we are unaware of literature which shows how each treatment approach affects co-ocurring comorbidities differently and how patients may uptake different treatments based on comorbidities. To better account for patient heterogeneity, the model could be updated with real-world patient data when available, and this may improve the applicability of findings to clinical practice.

We did not account for the additional cost of comorbidities in those with GAD. This was because it is difficult to separate out treatment effects for those with GAD only and those with GAD and comorbidities from the literature. The Markov framework is only as good as the parameters used to calibrate it and we are unaware of quality estimates (i.e., those generated from randomized controlled trials) that address the impact of each individual treatment option on further sample populations, including those with comorbid conditions and/or GAD in the presence of comorbid conditions to include in the model. The assumed parameters may not reflect people from different health backgrounds or diverse populations, responsiveness to treatment, or the impact on people who work in lower paid and less flexible occupations. We did not assume the individual cost of lost wages and time away from work to attend treatment-related appointments because of our focus on a payer and societal perspective. Findings may not be generalizable to other digital CBT platforms because our results are based on direct evidence from one standardized treatment from one randomized clinical trial [28]. Further research testing of this digital CBT treatment is underway (ClinicalTrials.gov ID: NCT05748652), and results suggest similar treatment effects to those presented here [32]. It may be helpful to note that independent researchers have reviewed this trial and have found to it to be of a low-risk of bias [67]. Readers may also use the charts to better understand in what way rates of remission (Figs 2 and 3) and attrition (Figs 6 and 7) impact the NMB of digital CBT relative to further treatments. This Markov model did not make assumptions regarding patient treatment preferences surrounding treatment selection because of the difficulty quantifying this as a model input. These limitations are common in a simulation exercise and adding model complexity is likely to strengthen the overall finding that digital CBT is a cost-effective treatment for GAD. Further researchers may wish to verify our approach by running similar models, with inputs documented in Table 1, and systematic reviews once further results are avaliable. Lastly, we also assumed slightly higher rates of remission than may be expected for individual and group CBT, and pharmacotherapy treatments in order to be conservative in our evaluation for when comparing with digital CBT.

## Conclusion

In both a payer and societal perspective, fully automated digital CBT is a cost-effective and cost-beneficial treatment for GAD in the US. Over a 12-month time horizon, the cost of treating GAD with digital CBT was lower than the cost of not treating GAD (i.e., a do-nothing scenario). Digital CBT is cost-effective because it reduces GAD-related healthcare and work-related costs. Relative comparisons to alternative treatments and to no treatment reveal that digital CBT generates the most value. Digital CBT for GAD is a cost-effective solution when the intention is to provide access to CBT at a population scale.
